# Biological Parenthood in Relation to Future Artificial Reproductive Technologies: A Scoping Review of Definitions

**DOI:** 10.1007/s11673-025-10520-w

**Published:** 2026-03-13

**Authors:** Lisa A. van Roermund, J. J. Jos Kole, Marijke J. ter Voert, Machteld J. Vonk

**Affiliations:** 1 Faculty of Law, Radboud University, Montessorilaan 10, Nijmegen, Gelderland 6525 HR The Netherlands; 2 Radboud University Medical Center – IQ Health, Kapittelweg 54, Nijmegen, Gelderland 6525 EP The Netherlands

**Keywords:** Biological parenthood, Ectogenesis, In vitro gametogenesis, Mitochondrial replacement therapy, Scoping review

## Abstract

Future artificial reproductive technologies, including mitochondrial replacement therapy, in vitro gametogenesis, and ectogenesis raise important legal and ethical questions, such as: Who should be considered the parents of children born from these technologies? The concept of biological parenthood is commonly used as a basis for answering this question. Therefore, this study assesses the use and meaning of biological parenthood in relation to these future artificial reproductive technologies. Searches were conducted in six databases, including PubMed, PsycINFO, Web of Science, HeinOnline, Westlaw Classic, and Philosopher’s Index. Articles were included only if they met one of the following conditions: 1) a definition of biological parenthood, 2) a definition of genetic parenthood, 3) a definition of gestational parenthood, 4) a definition of biological, genetic, or gestational parenthood combined with either mitochondrial replacement therapy, in vitro gametogenesis, or ectogenesis. Of the 6,304 studies retrieved, 131 results were included for analysis. The review found multiple meanings of biological parenthood, encompassing different biological ties (genetic, gestational, and postnatal) and diverse conceptual methods of interpreting its meaning. The definition of gestational parenthood appears to be less univocal than the term initially suggests, and that of genetic parenthood has evolved. The lack of consensus on the meaning and definition of biological parenthood leads to inconsistent determinations of biological parenthood in relation to future artificial reproductive technologies. This raises serious doubts about whether biological parenthood can continue to play the fundamental role in ethical and legal perspectives on parenthood that it has held for a long time.

**Supplementary Information:**

The online version contains supplementary material available at https://doi.org/10.1007/s11673-025-10520-w.

## Introduction

### Background

Artificial reproductive technologies (ARTs) increase the likelihood of achieving parenthood but also raise questions about the definition—and even the very concept—of biological parenthood, which is usually considered the moral and legal basis for defining parenthood as such (Fuscaldo 2003; Piotrowska 2018). Before the advent of ARTs, the concept of biological parenthood was undisputed. The gestational mother was always one of the child’s two genetic contributors, and thus the concept of biological parenthood was synonymous with genetic parenthood (Junewicz and Billick 2018). With the development of ARTs such as surrogacy, gamete donation, embryo freezing, and in vitro fertilization (IVF), children can have multiple biological origins (Bayne and Kolers 2003). For example, high-tech surrogacy has made it possible for a child to have genetic parents (gamete providers, who supply the sperm of the egg) and a separate gestational parent (the surrogate mother) (D’Alton-Harrison 2014). Consequently, ARTs have fragmented the concept of biological parenthood into diverse components.

While currently available ARTs already challenge the concept of biological parenthood and its foundational role in ascribing parenthood, future ARTs are expected to complicate this concept and its role even further. Examples of future ARTs include mitochondrial replacement therapy (MRT), in vitro gametogenesis (IVG), and ectogenesis. MRT is already in use, and IVG and ectogenesis are expected to be used on humans in the near future (McMillan 2021). These technologies may provide novel methods for biological relations between children and parents, including the use of genetic material from multiple individuals and gestation without the involvement of a human body (Bowman-Smart 2021).

Given the advanced development of MRT, IVG, and ectogenesis, along with their implications for parenthood, a pertinent question is: “how will parenthood be defined with respect to children resulting from these future ARTs, and what role can the presumed fundamental concept of biological parenthood still play in this regard?”.

Despite the amount of literature on ARTs, the impact of future ARTs on biological parenthood is understudied (Hendricks 2006). In addition, a systematic exploration of definitions of biological parenthood in relation to future ARTs is still lacking.^1^ A scoping review provides a multifaceted lens to map the existing literature within a particular domain systematically and transparently (McDougall 2015; Parsons and Johal 2022). Therefore, we conducted a scoping review to examine how biological parenthood was used in discussions about the parenthood of children resulting from future ARTs.

Biological parenthood is usually considered fundamental in understanding and ascribing parenthood in ethics and law. According to Piotrowska (2018), without an understanding of the concept of biological parenthood, our discussions on parent–child relationships and the ethical dilemmas they pose lack a principled basis. Bowman-Smart (2021) contends that a coherent conceptual framework for biological parenthood enables individuals to navigate the significant relationship between parents and children and clarifies the moral and legal obligations it entails, such as parental rights and obligations towards their children. Consequently, when the concept of biological parenthood is unclear, it leads to ethical and legal uncertainty regarding who holds parental rights and obligations on this basis. Such uncertainty may negatively affect both (prospective) parents and (future) children. Rights serve to protect vulnerable individuals, such as children, while obligations ensure that designated individuals, such as parents, are held responsible for their care. In many legal systems, the foundational role of biological parenthood has traditionally been reflected in legal definitions of parenthood (Hendriks et al. 2017). For instance, the gestational mother is automatically designated as the legal mother of the child (Gruenbaum 2012).^2^ This legal recognition stems from a biological reality that the gestational mother is also the genetic mother, and ensures that someone is immediately responsible for the care of a child (Hendriks et al. 2017; Palazzani 2020).

This review is intended to provide both a starting point for future research and recommendations for a regulatory framework governing parentage law in the Netherlands that is future-proof and can adapt to advances in ARTs. The key question in this regard is “how can legal parenthood be defined in relation to children born through ARTs?” As with biological parenthood, research on the impact of ARTs on legal parenthood is currently limited (Margalit et al. 2014). Given that a strong connection between biological and legal parenthood is still commonly assumed, this review provides insights that can inform the development of a future legal framework for assigning legal parenthood.

### Review Questions and Terminology

The review focuses on two key questions. The first research question is “what definitions of biological parenthood exist in the literature of many academic disciplines, including law, medicine, psychology, (bio)ethics, sociology, and philosophy?”^3^ The second research question is “what gaps, overlaps, limitations, and differences exist in the definitions of biological parenthood in relation to future ARTs, specifically MRT, IVG, and ectogenesis?” Table 1 defines the key terms applied in this review.

**Table 1:** Terminology

Term	Definition
Mitochondrial replacement therapy (MRT)	Mitochondrial replacement therapy (MRT) aims to prevent the maternal transmission of mitochondrial DNA (mtDNA) diseases. These diseases can lead to severe health issues, such as deafness, blindness, and muscular dystrophy (Baylis 2013) MRT is an IVF technique in which the affected mitochondria are removed from the egg and replaced with mitochondria from a healthy donor egg. As a result, the mitochondrial donor adds a small percentage to the child’s DNA (less than 0.1 per cent) (Baylis 2013). In addition, MRT encompasses various techniques, such as maternal spindle transfer (MST), and pronuclear transfer (PNT). The main difference between MST and PNT is timing: in MST, the mitochondria are exchanged before fertilization, whereas in PNT the exchange takes place after fertilization (Rulli 2017) Currently, the United Kingdom (from 2015) and Australia (from 2021) are the only countries with legislation specifically addressing clinical MRT use (Noohi et al. 2022). Consequently, in this review MRT is considered a “future ART” because of the absence of regulatory frameworks in other countries, including the Netherlands
In vitro gametogenesis (IVG)	In vitro gametogenesis (IVG) is a method that facilitates the production of gametes from adult somatic cells, stem cells, or progenitor cells outside the body (in vitro) (Hendriks et al. 2015). Its application in clinical settings for humans is still considered experimental (Notini et al. 2020) Stem cells can be sourced from various origins. Firstly, induced pluripotent stem cells (iPSCs) can be utilized to generate in vitro gametes. This process involves the genetic reprogramming of a somatic cell (such as a skin cell) from one of the prospective parents to create iPSCs. These iPSCs are then transformed into in vitro gametes (IVG gametes), which can be combined with other IVG gametes or non-IVG gametes in a laboratory setting. Secondly, IVG gametes may also be produced from embryonic stem cells (ESCs) obtained from cloned embryos created through somatic cell nuclear transfer (SCNT). Thirdly, IVG gametes can be derived from ESCs obtained from embryos created through in vitro fertilization (IVF) (Notini et al. 2020) IVG could potentially be applied for “solo reproduction,” where a single individual undergoes IVG to produce either male or female gametes. This method can be appealing to individuals without a partner or those who wish to have children independently. Here, IVF could be applied to fertilize the eggs of a single female with sperm derived from IVG or the sperm of a single male could fertilize eggs derived from IVG (Suter 2016)
Ectogenesis	Ectogenesis is the process of gestation of a developing embryo or fetus outside the human body, for example with artificial womb technology. Currently, it is an experimental technology for human application (McMillan 2021) Ectogenesis could either be done partially or completely. The term “partial ectogenesis” encompasses two distinct interpretations. From one perspective, it involves transferring a partially developed embryo or fetus from the female body to an artificial womb for the remaining gestational phase. On the other hand, it also refers to existing neonatology practices, like incubators, that support preterm infants and reproductive medicine techniques like IVF. The term “complete ectogenesis” denotes a scenario in which the entire gestation process, from fertilization to birth, occurs entirely outside the human body (Segers 2021)
Parenthood	Parenthood is associated with having parental obligations and responsibilities (Bowman-Smart 2021). Nowadays, parenthood encompasses various concepts, including legal, moral, social, intentional, and biological parenthood (Meyer 2006). To encompass the broader spectrum of individuals who can contribute to procreation through future ARTs, the term “parenthood” is consistently applied throughout this review, replacing the gendered terms “motherhood” or “fatherhood” (Gheaus 2018)

## Materials and Methods

### Search Strategy

To investigate the concept of biological parenthood in relation to future ARTs, a scoping review was carried out in accordance with Preferred Reporting Items for Systematic Review and Meta-Analysis Protocols (PRISMA-P) and the PRISMA extension for Scoping Reviews (PRISMA-ScR) (Tricco et al. 2018).

An initial search of PubMed and PsycINFO yielded a preliminary list of relevant articles. The titles, abstracts, and index terms from these sources were collected to develop a comprehensive search strategy for PubMed, PsycINFO, Web of Science, HeinOnline, Westlaw International, and Philosopher’s Index. These databases offer scholarly resources across a wide range of academic disciplines, including legal, medical, philosophical, sociological, and psychological research. The search strategy was developed and tailored in consultation with an experienced librarian at Radboud University Medical Center in Nijmegen, the Netherlands. The searches were conducted on September 22, 2023. In addition, a manual search of the reference lists of included studies was conducted to identify further relevant studies. No date limits were applied.

Search terms included all potential keywords related to biological parenthood, genetic parenthood, gestational parenthood, IVG, and ectogenesis. We deliberately excluded search terms related to MRT because this technology is referred to by numerous alternative names (Ravitsky et al., 2015). Since our searches already yielded multiple relevant results for MRT, adding all possible names would have generated noise and reduced the precision of our findings for the other research topics. Whenever feasible, medical subject heading (MeSH) terms were utilized in PubMed, and Index terms were employed in APA PsycINFO via Ovid. Table 2 presents the specific search term combinations applied in each database (“search key”) and when the searches were conducted (Indekeu et al. 2021).

**Table 2:** Search keys applied in each electronic database

Database	Search keys
PubMed	SEARCH KEY concept 1: (((“Reproductive Techniques”[Mesh:NoExp] OR “Reproductive Techniques, Assisted”[Mesh] OR “Ectogenesis”[Mesh] OR “Gametogenesis”[Mesh] OR ectogenesis[tiab] OR gametogenesis[tiab] OR “in vitro generated gametes”[tiab: ~ 1] OR gestational*[tiab] OR reproductive technol*[tiab]) AND (“Parents”[Mesh] OR parent*[tiab] OR “Family"[Mesh:NoExp] OR “Parent–Child Relations”[Mesh] OR “Parenting”[Mesh])) OR biological parent*[tiab] OR genetic parent*[tiab] OR gestational parent*[tiab] OR biological mother*[tiab] OR gestational mother*[tiab] OR genetic mother*[tiab] OR (genetic identity[tiab] AND child*[tiab])) combined with SEARCH KEY concept 2: (“Bioethical Issues"[Mesh] OR “ethics” [Subheading] OR “Concept Formation”[Mesh] OR concept*[tiab] OR defini*[tiab] OR redefin*[tiab] OR moral[tiab] OR morally[tiab] OR critical*[tiab] OR account*[tiab] OR debate[tiab] OR “address the problem”[tiab: ~ 3] OR critique[tiab] OR deliberat*[tiab])
APA PsycINFO via Ovid	SEARCH KEY concept 1: (((reproductive technology/ or epigenetics/ OR ectogenesis.ti,ab,id. OR gametogenesis.ti,ab,id. OR in vitro generated gametes.ti,ab,id. or reproductive technol*.ti,ab,id.) AND (parents/ or exp fathers/ or exp mothers/ OR parent*.ti,ab,id. OR family/ or biological family/ or "family of origin"/ or nuclear family/ OR parent child relations/ or father child relations/ or mother child relations/)) OR biological parent*.ti,ab,id. OR genetic parent*.ti,ab,id. OR gestational parent*.ti,ab,id. OR biological mother*.ti,ab,id. OR gestational mother*.ti,ab,id. OR genetic mother*.ti,ab,id.) combined with SEARCH KEY concept 2 (bioethics/ OR concepts/ OR concept.ti,ab,id. or concepts.ti,ab,id. or conceptualis*.ti,ab,id. or conceptualiz*.ti,ab,id. OR defini*.ti,ab,id. OR redefin*.ti,ab,id. OR moral.ti,ab,id. OR morally.ti,ab,id. OR critical*.ti,ab,id. OR account*.ti,ab,id. OR debate.ti,ab,id. OR ethics.ti,ab,id.)
Web of Science (Core Collection)	SEARCH KEY concept 1 TS = ((((“reproductive technol*” OR epigenetics OR ectogenesis OR gametogenesis OR “in vitro generated gametes”) AND (parents OR parent OR parenthood OR parenting)) OR “biological parent*” OR “genetic parent*” OR “gestational parent*" OR “biological mother*” OR “gestational mother*” OR “genetic mother*”) combined with SEARCH KEY concept 2 (Bioethic* OR ethics OR concept OR concepts OR conceptualis* OR conceptualiz* OR defini* OR redefin* OR moral OR morally OR critical* OR account* OR debate))
HeinOnline (Law Journal Library)	SEARCH KEY concept 1 (((“Reproductive techn*” OR epigenetics OR ectogenesis OR in vitro generated gametes OR gametogenesis) AND (“biological parent*” OR “genetic parent*” OR “gestational parent*” OR “biological mother*” OR “gestational mother*” OR “genetic mother*”) AND (Bioethic* OR defini* OR redefini* OR moral* OR critical* OR account* OR debate)))
Westlaw Classic (International Materials)	SEARCH KEY concept 1 adv: (((“Reproductive technol!” OR epigenetics OR ectogenesis OR in vitro generated gametes) & (“biological parent!” OR “genetic parent!” OR “gestational parent!” OR “biological mother!” OR “gestational mother!” OR genetic mother!”) & (Bioethic! OR defini! OR redefini! OR moral! OR critical! OR account! OR debate)))
Philosopher’s Index via Ovid	SEARCH KEY concept 1 (((reproductive technology.mp. or epigenetics.mp. OR ectogenesis.mp. OR gametogenesis.mp. OR in vitro generated gametes.mp. or reproductive technol*.mp.) AND (father*.mp. or mother*.mp. OR parent*.mp. OR family.mp.)) OR biological parent*.mp. OR genetic parent*.mp. OR gestational parent*.mp. OR biological mother*.mp. OR gestational mother*.mp. OR genetic mother*.mp.) combined with SEARCH KEY concept 2 (bioethics.mp. OR concepts.mp. OR concept.mp. or concepts.mp. or conceptualis*.mp. or conceptualiz*.mp. OR defini*.mp. OR redefin*.mp. OR moral.mp. OR morally.mp. OR critical*.mp. OR account*.mp. OR debat*.mp. OR ethics.mp.)

### Study Selection

The review only included studies that address one of the following criteria: 1) a definition of biological parenthood, 2) a definition of genetic parenthood, 3) a definition of gestational parenthood, 4) a definition of biological, genetic, or gestational parenthood combined with either MRT, IVG, or ectogenesis. There were no restrictions on publication dates.

The review also included studies with definitions of biological motherhood and biological fatherhood. Given the gender-neutral language applied in this article, there will be no further distinction between biological motherhood and fatherhood. Nevertheless, these studies were included to provide a comprehensive understanding of biological parenthood. Moreover, the review also incorporated studies that addressed definitions of genetic and gestational parenthood.

Furthermore, this review focused on three future ARTs: MRT, IVG, and ectogenesis. These technologies were selected for analysis because they present a unique set of challenges with regard to the attribution of parenthood. Consequently, they provide a valuable point of departure for ethical and legal reflection on the assignment of parenthood. In addition, the review focused on MRT and ectogenesis in general, without delving into specific applications of these ARTs. However, for IVG, the review specifically included results referring to the utilization of induced pluripotent stem cells (iPSCs) for both couple and single-person reproduction (solo reproduction), as well as the application of embryonic stem cell-derived gametes (ESCs) created through IVF. This decision was made to delineate the scope of the review.

Only journal articles were included from the database searches in the review. The review did not include works such as books and book chapters, book reviews, commentaries, dissertations, and blogs. Additionally, the review only included works that were published in English and Dutch because of the reviewer’s language proficiency in these two languages.

### Screening

Articles identified from database searches were collated and uploaded in Endnote v.20.01 (Clarivate Analytics, PA, USA) and duplicate articles were subsequently removed. A pilot test of the screening and selection process was undertaken with a reviewer (L.v.R.) and a verifier on a random sample of fifty articles to ensure a consistent interpretation of the eligibility criteria. Following the pilot test, title and abstract screening were conducted in Rayyan online software (Qatar Computing Research Institute, Doha, Qatar) (Ouzzani et al. 2016). Initially, one reviewer (L.v.R) independently screened titles and abstracts against the inclusion and exclusion criteria to identify potential eligible articles. To maintain consistency and validate the reviewer’s (L.v.R.) assessment, the verifier rescreened a random sample of 20 per cent of the titles and abstracts. The limit of 20 per cent was set in accordance with the available time and resources. Any disagreements about whether to include or exclude studies were resolved through consensus reached via discussion. The Cohen’s kappa inter-rater reliability score was 0.98, indicating excellent agreement between the reviewer and the verifier.

Following this title/abstract screening, articles deemed eligible for full-text screening were examined by a reviewer (L.v.R.) against the inclusion and exclusion criteria. Reasons for exclusion were documented and reported in the review. Subsequently, another verifier (M.V.) rescreened 20 per cent of the full-text articles before final inclusion in the review. The reasons for excluding sources after reviewing the full-text were recorded and reported in the review. Any disagreements about whether to include or exclude studies were resolved through consensus reached via discussion. The Cohen’s kappa inter-rater reliability score was 0.78, indicating substantial agreement between the reviewer and the verifier.

**Fig. 1:**
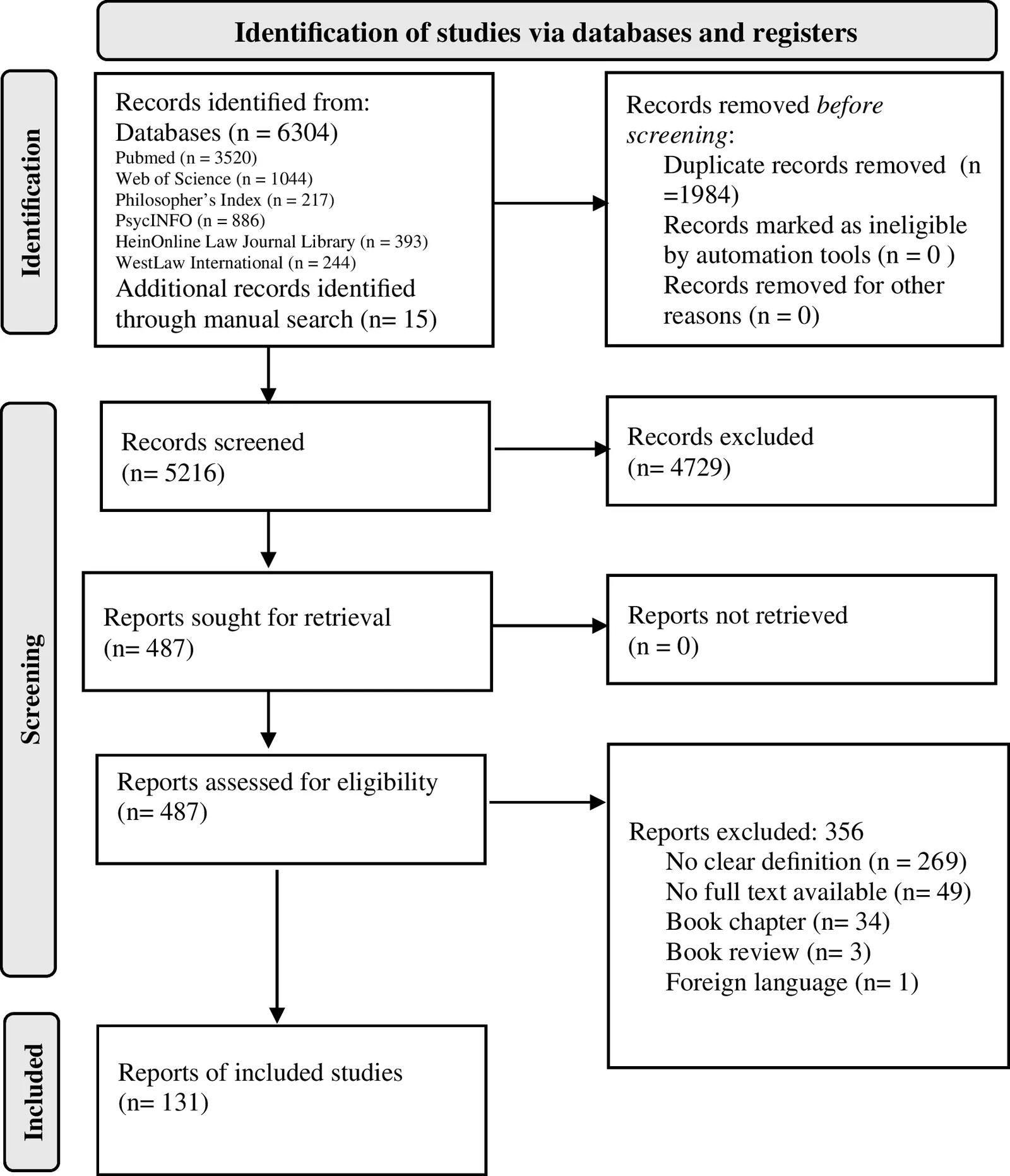
PRISMA flow diagram

The results of the search and the screening process are reported in full in the review and presented in a Preferred Reporting Items for Systematic Reviews and Meta-analyses extension for Scoping Reviews (PRISMA-ScR) flow diagram (Fig. 1) (Tricco et al. 2018).

### Data Extraction

Data from articles were extracted by a reviewer (L.v.R.) using a self-developed data extraction tool based on the Joanna Briggs Institute (JBI) Evidence Synthesis Extraction Template (Aromataris et al. 2024). The data extraction tool was made in a Microsoft Excel file (Redmond, Washington, USA). During extraction, the following information was charted: author details, publication year, title, journal, database, language, and key findings relevant to the review questions. This article presents (sections of) tables containing data extracted from the included studies. The full data extraction form is included in Supplement I.

## Results

### Search Results

The databases yielded 6,304 publications, of which 1,984 were duplicates. After applying the inclusion and exclusion criteria, 487 articles were left. These articles were further evaluated by screening the full text. During the full-text screening stage, 356 articles did not meet the inclusion criteria for this review. Eventually, 131 eligible studies were included in the scoping review (Fig. 1).

### Review Findings

This review focuses on definitions of biological parenthood and on what gaps, overlaps, limitations, and differences exist in these definitions in relation to future ARTs, specifically MRT, IVG, and ectogenesis. The review findings will be presented in three thematic sections. First, the findings on biological parenthood will be discussed. Since these findings indicate that biological parenthood primarily encompasses genetic and gestational parenthood, findings on these types of parenthood will also be addressed in separate thematic sections. Subsequently, the review findings will be considered in relation to future ARTs.

#### Biological Parenthood

The review revealed a predominant focus on genetic and gestational ties in the definitions of biological parenthood (see Supplement I). For instance, Zeiler and Malmquist described biological parenthood as follows: “The term biological parenthood commonly refers to genetic and/or gestational parenthood and we will follow this usage” (2014, 348).

However, while genetic and gestational connections are commonly discussed in the context of biological parenthood, the review also identified some alternative biological connections between parents and children and diverse views on how to define the meaning of concepts. For example, Ettinger (2012) argued that there is too much emphasis on purely genetic approaches in the concept of biological parenthood. Instead, biological parenthood should be regarded as a “family resemblance concept,” meaning that there are no necessary and sufficient conditions that every biological parent must always meet to count as a biological parent. Instead, how biological parenthood is defined depends (according to Ettinger’s interpretation of “family resemblance”) on the paradigm of biological parenthood used. For Ettinger, this entails that a particular type of biological parenthood is used as a paradigm to define biological parenthood in general. The specific characteristics of that particular type of biological parenthood determine how future cases are considered. Biological parenthood in such cases is recognized to the extent that it is sufficiently similar to the characteristics of the type of parenthood taken as a paradigm.

Just like Ettinger (2012), Maung (2021) pointed out that the biological tie is restrictively interpreted (namely, in purely genetic terms), overlooking the impact of childrearing interactions, like feeding, bodily contact, sensory stimulation, and positive reinforcement. These interactions can have lasting biological effects, potentially leading to resemblances and shaping the children’s identities. Furthermore, Junewicz and Billick (2018) similarly viewed biological parenthood as a comprehensive concept that includes the psychological parent. They define a “biological parent” as the parent with whom the child shares a strong attachment bond. Attachment, in this context, is understood as a biological-psychological process occurring in the brain, influenced by environmental factors.

The variety of biological connections is also apparent in children’s descriptions of their biological parents. In a study by Raes et al. (2015), qualitative research was conducted involving in-depth interviews with six children, aged nine to ten, from lesbian families, all conceived via anonymous sperm donation. The study aimed to explore family and parental concepts among children raised in lesbian households. Through semi-structured interviews, the researchers analysed the children’s comprehension of the roles of the biological mother, non-biological mother, and sperm donor in shaping their families. When asked to define “biological mother,” the children generally mentioned two primary characteristics: caregiving for the child (such as preparing food and buying clothes) and a biological/causal link, although the distinction between gestational, genetic, and causal links was not always clear.

Ultimately, biological parenthood is not only defined in terms of genetic and gestational parenthood. In addition, non- or less-reductionist interpretations of “biological” have been found.

#### Gestational Parenthood

The review demonstrated, as the term itself implies, that the definitions of gestational parenthood mainly focus on gestation and childbirth (see Supplement I). For instance, Bayne argued that “gestationalists claim that parenthood is based on gestation and childbirth” (2003, 78).

Besides referring to gestation and childbirth, some results added further subtleties to the concept of gestational parenthood. For instance, both Dury (2016) and Black (2018) cited Lady Hale, who mentioned conceiving the child as part of gestational parenthood. This excludes, for example, situations where someone carries an embryo created through IVF, since the gestational carrier would not be involved in the conception. Furthermore, Ber (2000) and Zafran (2008) incorporated a temporal element in their definitions. They define gestational parents as those who carry the child “to term” or for “nine months” respectively (Ber 2000, 154; Zafran 2008, 117). This may suggest that gestational parenthood may necessitate a full-term pregnancy, that could exclude premature births.

Ultimately, gestational parenthood is primarily defined by gestation and childbirth, although some definitions include additional subtle nuances.

#### Genetic Parenthood

The review showed diverse definitions of genetic parenthood (see Supplement I). Table 3 presents a chronological overview of proposed definitions of genetic parenthood, as the results of this review have shown that definitions build on each other. To avoid redundancy, results referring to the same definitions have been combined. Where available, the original titles of the definitions are included. In cases where a definition lacks a specific title, the author’s surname is used.

**Table 3:** Chronological overview of genetic parenthood

Author and year	Name of the definition or, if this is not available, last name of the author(s)	Definition of genetic parenthood
Multiple authors (for example, Bayne and Kolers 2003)	**Direct Derivation Account (DDA)**	A genetic parent is someone who is a direct genetic source of a child
Multiple authors (for example, Mertes 2014)	**Informational Overlap (IO)**	[A] child is my genetic child when it has 50 per cent of my DNA or when it has twenty-three of my chromosomes OR P is C’s genetic parent if and only if 50 per cent of C’s genetic material encodes the same information as 50 per cent of P’s DNA (Douglas and Devolder 2019)
Griesemer 2000	**Griesemer**	The process of reproduction can be analysed as multiplication of material overlapping propagules that confer the capacity to develop, specified in terms of the minimum notion of development as acquisition of the capacity to reproduce
Mertes and Pennings 2008	**Mertes and Pennings**	X is considered a genetic parent of Y if (i) X passes on genetic information to Y, (ii) X is a physical cause of Y’s existence, and (iii) X’s physical genes were reshuffled once to constitute Y
Ettinger 2012	**Pure Genetic Approach (PGA)**	Conceptus must share some of the parent’s genetic makeup and the parent must somehow be causally responsible for it
Ettinger 2012	**Weak Genetic Approach (WGA)**	Any genetic contribution
Ettinger 2012	**The Degree Theoretic Approach (DTA)**	One is a biological parent to the same degree that one contributes genetic information to another
Ettinger 2012	**Genetic Threshold Approach 50% (GTA)**	Threshold of genetic donation; natural cut-off point is 50 per cent contribution of DNA
Douglas 2014	**Direct Proportionate Genetic Descent (DPGD)**	P is the genetic parent of C if and only if (a*) C inherited some (or perhaps some specified proportion) of his or her genetic material from P, and (b*) the genetic material was not transmitted from P to C via another *human being*
Moschella 2014	**Moschella (slightly modified definition per Mertes and Pennings)**	A is a genetic parent of B if (i) A passes on genetic information to B, (ii) A’s physical genome is passed on to B, and (iii) A’s physical genome was reshuffled once (usually mixed with the genome of a different individual) to constitute B
Palacios-González 2018	**Modified Direct Genetic Derivation (MDGD)**	The parental genetic link that exists between A and B grounds parental responsibilities if the act that gave rise to the genetic derivation was one that B consented to in an informed way
Piotrowska 2018	**Overlap, Development and Persistence (ODP)**	Material overlap between parent and offspring; the inherited parts contribute to the development of the offspring's own reproductive capacities and the material parts, or their descendants, are passed down to the next generation
Mertes 2014, summarized by Douglas and Devolder 2019	**Direct Genetic Descent (DGD)**	P is C’s genetic parent if and only if (a) C’s genes are derived from P’s genes, and (b) not through deriving from the genes of some third, intervening individual, M
Douglas and Devolder 2019	**Direct Gametic Genetic Descent (DGGD)**	P is C’s genetic parent if and only if (a) C’s genes are derived from P’s genes, (b) through a gamete produced by P, and (c) not through deriving from the genes of some third, intervening individual, M
Douglas and Devolder 2019	**Modified Direct Proportionate Genetic Descent (MDPGD)**	P is C’s genetic parent if and only if (a) some proportion X of C’s genes are derived from P’s genes and (b) not through deriving from the genes of some third, intervening individual M from whom C derived proportion Y of his genes
Simkulet 2021	**Substantive Historical Direct Genetic Descent (SGD)**	P is C’s genetic parent if and only if a substantive portion of C’s genes are derived directly from P’s genes

Traditionally, a simple understanding of genetic parenthood prevailed, based on the assumption that each person contributes 50 per cent of the child’s DNA (Cavaliere and Palacios-González 2018). Several authors considered this definition too broad, as it could also include siblings—who would also share around 50 per cent of DNA—as genetic parents (Palacios-González 2018). Moreover, many genes are common so, in principle, someone else could share half a child’s DNA purely by coincidence (Sparrow 2006). In addition, nearly all human DNA is identical (99.9 per cent), which makes the 50 per cent benchmark insufficient for defining genetic parenthood (Douglas and Devolder 2019).

To address these limitations, several authors proposed definitions including direct genetic derivation. According to Mertes (2014, 744), who referred to Kolers (2003), direct genetic derivation entails: “X is a genetic child of Y if X is directly derived from Y’s genes.” The inclusion of “derivation” in this definition serves two key purposes. First, it establishes a distinction between parents and other blood relatives, such as siblings, who may share DNA with a child without being directly involved in the child’s genetic makeup. Second, it helps differentiate parents from grandparents, as grandparents contribute their genetic material indirectly through their children (Mertes 2014).

Moreover, the concept of genetic derivation involves two aspects: the physical transmission of genes and the causal relationship with the offspring (Bayne and Kolers 2003; Douglas 2014; Douglas and Devolder 2019; Mertes 2014; Palacios-González 2018; Simkulet 2021). For instance, Kolers’ (2003) Physicalistic Geneticism (PG) emphasized the informational role of DNA, recognizing the physical nature of humans and reproduction. Sparrow (2006) further highlighted the causal connection, arguing that genetic relatedness moves beyond recognizing shared genes to considering how those genes were acquired (Sparrow 2006; Piotrowska 2018).

Several authors criticized the concept of genetic derivation and proposed additional criteria or alternative definitions to accommodate the concept of genetic parenthood in diverse situations. Palacios-González (2018) argued that a child simply inheriting genes is not sufficient for determining genetic parenthood. He used a scenario of stolen gametes at an IVF clinic, where someone becomes a genetic parent without their consent. Therefore, Palacios-González (2018) proposed to add a requirement of informed consent to address this issue. Building on Kolers’ (2003) and Sparrow’s (2006) work, Mertes and Pennings (2008) suggested an additional criterion for genetic parenthood: the reshuffling of physical genes during inheritance from parent to offspring. This concept removes the requirement for direct genetic derivation, specifically to exclude situations like cloning (Mertes and Pennings 2008; Piotrowska 2018). Later, Moschella (2014) refined this concept of reshuffling in her definition, adding that the physical genome is typically mixed with another individual’s genome during reproduction.

Various scholars have commented on Mertes and Pennings’ (2008) definition, which includes the reshuffling of physical genes. For example, Piotrowska (2018) stated that the definition is too restrictive since it excludes reproduction cases without genetic reshuffling, such as MRT. Therefore, she proposed the Overlap, Development, and Persistence (ODP) account, based on Griesemer’s account (Griesemer 2000). On the ODP account, genetic parenthood can be established through material overlap, phenotypic contribution, and intergenerational persistence. The first two criteria entail sharing genetic material with the offspring and contributing to the offspring’s ability to reproduce, corresponding with Griesemer (2000). According to Piotrowska (2018), these two requirements alone are not well suited for assisted reproduction, as they do not specify how and to what extent transferred parts must be linked to the recipient’s ability to reproduce. This could lead to situations where simply sharing any genes (even non-reproductive ones) qualifies as genetic parenthood. Therefore, Piotrowska (2018) added the requirement of intergenerational persistence, which necessitates that the heritable elements necessary for reproduction must be passed on to the next generation.

Douglas and Devolder (2019) rejected the account of Piotrowska (2018) and argued that material overlap is not an essential requirement for genetic parenthood. Even if sperm transmits genetic information without physical contact, a genetic parent–offspring relationship still exists between the sperm donor and the resulting offspring. The authors started by (re)formulating Mertes’ (2014) concept of direct genetic derivation: “P is C’s genetic parent if and only if (a) C’s genes derived from P’s genes, and (b) not through deriving from the genes of some third, intervening individual, M” (Douglas and Devolder 2019, 56). Based on this concept, the authors explored various propositions for alternative definitions, ultimately favouring the Modified Direct Proportionate Genetic Descent (MDPGD) definition of genetic parenthood:

P is C’s genetic parent if and only if (a) some proportion X of C’s genes derived from P’s genes and (b) not through deriving from the genes of some third, intervening individual M from whom C derived proportion Y of his genes. (Douglas and Devolder 2019, 58)

While mentioning a 20–80 per cent range for illustrative purposes, they avoided specifying a fixed percentage for X and Y (Douglas and Devolder 2019).

The MDPGD account has been the subject of commentary by various authors. Palacios-González (2019) pointed out that the MDPGD account lacks a causality condition. This raises the possibility of someone becoming a genetic parent to an already-born individual, which contradicts the very essence of genetic parenthood. In addition, Bowman-Smart (2021) added several other objections to MDPGD, such as neglecting the genome’s role in favour of individual genes, lacking a defined threshold for an acceptable proportion of X and Y, and using the term “derived” ambiguously, as it is unclear if artificially derived sequences would constitute a derivation. While not offering a new definition, Bowman-Smart (2021) highlighted essential criteria for genetic parenthood: material overlap between genomes, a causal link between contributor and offspring, and some degree of resemblance. Moreover, Simkulet (2021) criticized the MDPGD account for being too restrictive. He argued that it fails to encompass scenarios like hybridization, cloning, and mitochondrial replacement therapy. Therefore, he proposed an alternative theory, the Substantive Historical Direct Genetic Descent (SGD) account, which is more flexible. Notably, SGD leaves room for debate about what constitutes a significant genetic contribution. Additionally, Simkulet (2021) argued that SGD can accommodate a variable number of parents, whereas Douglas and Devolder (2019) limit genetic parenthood to a maximum of two individuals.

Ultimately, the results showed no consistent definition of genetic parenthood. However, the definitions are continually evolving and building upon one another.

#### Biological Parenthood and Future ARTs

The review mapped out existing definitions of biological parenthood, including a focus on definitions of genetic and gestational parenthood.

This section will explore how the definitions of biological parenthood relate to future ARTs—specifically MRT, IVG, and ectogenesis—according to the reviewed literature. A distinct analysis will be conducted for each ART based on the available selected literature. Ectogenesis raises questions about gestational parenthood, and IVG and MRT challenge our understanding of genetic parenthood. For additional clarity, an overview of the definitions of genetic parenthood as applied to MRT and IVG is included in Supplement I, with “natural” sexual reproduction included for comparative purposes.

*Ectogenesis:* currently, all children have gestational parents, unless ectogenesis becomes available (Murphy and Parks 2020; Murphy 2017; Sparrow 2012). Ectogenesis has the potential to decouple the pregnancy from the human body and so to eliminate the gestational bond that typically forms between the person carrying the pregnancy and the developing fetus (Kennedy 2024; MacKay 2020). Despite the absence of gestational parenthood with ectogenesis, the possibility of genetic parenthood remains open (Kimberly et al. 2020).

In vitro* gametogenesis and induced pluripotent stem cells*: the application of induced pluripotent stem cells (iPSCs) raises relatively few questions about genetic parenthood, as it could encompass all the aspects discussed in the various definitions reviewed, such as material overlap, transmission of genetic information, reshuffling of genes, and causation. Therefore, there is a broad consensus in the literature on the existence of genetic parenthood in the application of iPSCs, since the “derived” gametes could achieve genetic parenthood in the same manner as “natural gametes” (Alghrani 2009; Carter-Walshaw 2019; Murphy 2014, 2017; Notini et al. 2020; Palacios-González et al. 2014).

In general, the application of IVG with iPSCs enables couples to conceive a child who shares approximately 50 per cent of the DNA from each parent (Segers et al. 2019). However, Segers et al. (2019) indicated that it is controversial whether it will be possible to produce gametes of the opposite sex and thus enable same-sex couples to have a child to which each partner makes an equal genetic contribution. However, this may not be a problem if a definition of genetic parenthood is applied that does not necessitate a 50 per cent contribution to total DNA (Segers et al. 2017).

In vitro* gametogenesis and embryonic stem cells obtained from IVF embryos:* in contrast to IVG using iPSCs, there is more discussion in the literature about IVG using embryonic stem cells (ECS) from embryos created by IVF concerning the concept of genetic parenthood (Notini et al. 2020; Sparrow 2012, 2014; Testa and Harris 2004). In this context, IVG would not be like conventional reproduction because the ECS are derived from embryos. With an increasing number of intended parents, each parent contributes a proportionally smaller share of their genes to the child. For example, quadruplets would each contribute 25 per cent and octuplets would contribute 12.5 per cent each, and so on (Notini et al. 2020; Suter 2016).

Debate exists within the literature regarding the temporal compression of generations, as the “middle generation” would essentially only be an embryo and the source of the gametes applied to produce the resulting child. Segers et al. (2017) and Suter (2016) argued that these contributors should be classified as grandparents rather than parents because the embryo adds another generation. Douglas (2014), meanwhile, regarded the embryo as a colony of human stem cells rather than a human being. Therefore, according to the Direct Proportionate Genetic Descent (DPGD) theory, users of this type of IVG are considered genetic parents since their genetic material was one of the inputs into the process and was not transmitted via another human being (Douglas 2014; Sparrow 2012). In contrast, Sparrow (2012) argued that children created via ECS would be considered “genetic orphans” since they would not have any living (genetic) parents, with their most immediate ancestors being now deceased embryos.

In vitro* gametogenesis and solo reproduction*: IVG also holds out the prospect of solo reproduction, where only gametes of one individual are used; namely, a “natural” gamete could be combined with a derived gamete from the same individual (Cutas and Smajdor 2017).

Compared to different future ARTs, solo IVG involves the counterintuitive possibility that, when applying certain definitions of genetic parenthood, a given child may not have a genetic parent at all. For example, under the Informational Overlap definition a child born by solo IVG has no genetic parent because one person shares 100 per cent of the child’s DNA instead of 50 per cent. Possibilities like this raise the question of what the implications are of not being able to identify a genetic parent at all. According to Suter, “depriving” children of a second genetic parent “raises serious red flags” (2016, 110). Furthermore, Suter states that this absence of a genetic parent could potentially undermine the relational autonomy of affected individuals by disrupting a common type of relational connection. Nonetheless, in such cases social parent relationships, which are also important for self-definition, remain a possibility.

*Mitochondrial replacement therapy:* in the literature, terms such as “three-parent babies,” “three-parent embryos,” and “three-person IVF” are often applied when discussing MRT (Baylis 2013; Carr 2015; Fischbach et al. 2014; Ishii and Palacios-González 2017; Johnson 2013; Mitalipov and Wolf 2014). This terminology implies that a mitochondrial donor is considered to be a genetic parent of the child. The question is whether these terms are accurate when applying definitions of genetic parenthood (Palacios-González 2018).

In the United Kingdom, the impact of MRT on genetic parenthood has already been assessed by several institutions. These institutions have concluded that a mitochondrial donor does not meet the criteria to be recognized as a genetic parent. For instance, the Department of Health argued that mitochondrial DNA (mtDNA) does not influence the personal characteristics of the child, because these sorts of genes come only from the nuclear DNA (Department of Health 2014; Dimond and Stephens 2018). In addition, the Nuffield Council on Bioethics mentioned the argument that the mitochondrial donor only adds a small percentage to the child’s DNA; namely, less than 0.1 per cent (Watts et al. 2012; Baylis 2013).

The stances taken by British institutions on this issue could be categorized into two approaches to genetic contribution: the quality (identity) and quantity (contribution to the total DNA) approach (Palacios-González 2018). Many definitions of genetic parenthood included in the review focus on the amount of DNA a person contributes. However, quantitative approaches to genetic parenthood result in inconsistencies concerning the genetic parenthood of the mitochondrial donor. For example, according to the Informational Overlap account, the mitochondrial donor is not considered a genetic parent, as the genetic contribution is less than 50 per cent of the total DNA (Mertes and Pennings 2008). In contrast, the Weak Genetic Approach (WGA) considers the mitochondrial donor as a genetic parent because of their genetic contribution, regardless of the amount of DNA (Ettinger 2012).

The dominant perspective in the literature suggests that only nuclear DNA determines our characteristics, although some studies question this assumption (Bredenoord et al. 2011). Furthermore, Baylis (2013) argued that personal identity is about more than appearances and character traits. Instead, Baylis (2013) proposed a narrative description of personal identity, suggesting that identity (including traits, desires, beliefs, etc.) is shaped not by genetic factors but by personal relationships that vary in degree and type of intimacy and interdependence. From this perspective, health and illness are also aspects of personal identity, so it would be incorrect to say that MRT does not contribute to a person’s identity (Baylis 2013). In addition, Bredenoord et al. highlighted that “a person born without a mitochondrial disease will have a different life experience, a different biography, and perhaps also a different character” (2011, 99) than they would if they had been born with such a condition. Thus, mtDNA is likely to change the (qualitative) identity of the child.

## Discussion

This article aimed to explore the definitions of biological parenthood in relation to future ARTs (MRT, IVG, and ectogenesis). A scoping review was used to map literature across a wide range of academic disciplines, using the databases PubMed, PsycINFO, Web of Science, HeinOnline, Westlaw International, and Philosopher’s Index. In total, 131 eligible studies were included in the scoping review.

The results indicate a lack of consensus on the concept of biological parenthood. This is reflected in the diversity of possible biological connections between parents and children mentioned in the literature. The traditional notion of a biological parent, defined solely by genetic contributions and gestation, has been significantly altered because of the introduction of ARTs. Genetic and gestational ties are, traditionally, the most frequently identified biological connections in definitions of biological parenthood. However, biological parenthood can also be interpreted broadly to include ties that may develop after birth, such as childrearing interactions and attachment (Maung 2021; Junewicz and Billick 2018; Raes et al. 2015).

Although genetic and gestational parenthood are meant to specify and clarify the broad term “biological parenthood,” it turns out that these parenthood concepts can themselves be interpreted in different ways. Even gestational parenthood appears to be less univocal than the term itself suggests concerning gestation and childbirth, with some definitions mentioning elements of conception and/or including temporal aspects. In addition, genetic parenthood has received considerable attention in the literature. The differing lengths of the three result sections of this article illustrates this. Such emphasis reflects, according to Maung, a “genocentric conception of the biological tie” (2021, 10), which posits genetic connection as the core element in defining biological parenthood. Moreover, the concept of genetic parenthood is constantly evolving, with newer definitions building upon and refining previous ones. This trend is likely to continue as new ARTs emerge.

Given their diverse interpretations, it may be questioned whether genetic and gestational parenthood can still be subsumed under the broad concept of biological parenthood. For instance, Douglas and Devolder (2019) stated that their concept of genetic parenthood differs from biological and causal parenthood and now coexists with these concepts, though it has largely replaced biological and causal parenthood in everyday discourse. This shift in conceptual hierarchy relativizes the foundational role of biological parenthood, as genetic and gestational parenthood, once encompassed within it, now emerge as independent parenthood concepts. Consequently, this raises doubts about whether biological parenthood can continue to serve as a fundamental basis for ethical and legal ascriptions of parenthood.

The results of this review also revealed a diversity of methods for defining the meaning of the key concept of biological parenthood. Ettinger (2012), drawing on the work of philosopher Ludwig Wittgenstein, argued that the definition of “biological parenthood” can be generated in multiple ways. One approach is essentialist, relying on identifying necessary and sufficient conditions for someone to be considered a biological parent. However, this approach could be problematic because—as it appears from this review as well—it is virtually impossible to establish a definitive list of necessary and sufficient conditions for defining biological parenthood (Hill 2017). Alternatively, biological parenthood can also be understood through the lens of so-called “family resemblance.” According to this view, all family members (all persons who are called parents) share some features with some other family members, but no family member has the same set of “essential” features that characterize all family members together (Ettinger 2012). Ettinger (2012) adds to this family resemblance approach the idea of an established paradigm of parenthood. A possible objection, however, to Ettinger’s family resemblance approach to the definition of “biological parenthood” is that what is considered close enough to the paradigm of biological parenthood is debatable and varies from person to person (Ettinger 2012). Besides, one might ask whether there could be one “paradigm family member” within a family. Appealing to an “established paradigm” seems to introduce an essentialist assumption that contradicts what the family resemblance approach wants to establish.

The diversity in methods for defining a concept (including essentialism and family resemblance approaches) might raise questions concerning the philosophical assumptions behind debates about the interpretation of biological parenthood. Many discussion partners in these legal and ethical debates appear to share an essentialist approach toward defining key concepts in common. A different approach to the definition of parenthood, whether biological, legal, or moral, and the corresponding definition of childhood—for example, through family resemblance—might open up promising avenues for discussing parenthood and childhood in relation to ARTs.

Differing definitions of biological parenthood, and differing methods for defining the meaning of this concept, eventually lead to inconsistencies regarding who is and is not recognized as a biological parent in the light of future ARTs (ectogenesis, IVG, and MRT). Consequently, this creates ambiguity in the interpretation of parent–child relationships and impacts ethical, moral, and legal discussions concerning who has parental rights and responsibilities (Bowman-Smart 2021). This definitional issue thus provides an additional reason to question whether biological parenthood can still serve its foundational role in ethical and legal accounts of parenthood.

There appears to be a common intuition that legal and moral parenthood cannot exist without reference to biological connections between parents and children. Illustrating this point, Meyer argued that “biology is increasingly called upon to share its privileged status as the foundation stone of parenthood with caregiving and other social values” (2006, 126). Given that the concept of biological parenthood has become fragmented in relation to future ARTs, biology seems less appropriate as a foundational basis for parenthood. In this vein, Ettinger (2012) contended that “biological parenthood” is primarily a descriptive concept based on common experiences, cultural biases, and prejudices, while “social parenthood” is a normative concept derived from biological parenthood. As the traditional biological understanding of parenthood fades with the advent of new technologies, the normative dimension of parenthood has become increasingly significant. Therefore, according to Ettinger, the normative concept of “social parenthood” requires closer attention, as it will play a crucial role in helping us navigate and understand emerging family structures. Furthermore, alternative accounts of parenthood have been proposed in the literature that do not emphasize biological ties. For instance, intentional and voluntarist perspectives on parenthood assign parental rights and responsibilities based on consent and the intention to provide social parenting, and thus prioritize the social, moral, and relational aspects of parenthood (Kendal 2023). Moreover, Bayne and Kolers argued that genetic relations can only ground parenthood within a pluralistic approach which entails that “any one of several kinds of relationship is sufficient for parenthood” (Bayne and Kolers 2003, 221).

Ultimately, this review is intended to provide both a starting point for future research and recommendations for a regulatory framework governing parentage law in the Netherlands that is future-proof and can adapt to advances in ARTs. The intersection of (future) ARTs with definitions of biological parenthood creates inconsistencies between the biological connections established by these ARTs and existing legal frameworks. For instance, the legal designation of the gestational mother would not automatically confer legal parenthood when a child is born from an artificial womb (Bayne and Kolers 2003). Moreover, most jurisdictions limit legal parenthood to a maximum of two individuals, reflecting the traditional notion of two biological parents (Abraham 2017).^4^ This limit of two legal parents is no longer consistent with the number of biological parents that could exist through ARTs. Therefore, scholars such as Kendal (2023) argue that legal systems should evolve to accommodate the scientific reality of having more than two biological parents. Because ascribing parenthood remains essential for assigning rights and obligations that protect children and hold parents accountable, new foundations for ethical and legal parenthood are needed.

## Strengths and Limitations

This study’s strength lies in its exploration of the gaps, overlaps, limitations, and differences in definitions of biological parenthood in relation to several future ARTs: ectogenesis, MRT, and IVG. Another key strength of this study is its methodological rigour, utilizing data from various research areas and databases. The review indicated that discussions of biological parenthood, in relation to future ARTs, occur in various scientific disciplines. However, it is notable that discussion of the concept of genetic parenthood is mainly prevalent within the bioethical literature, as compared to other scientific disciplines.^5^ A possible explanation is that, in the field of bioethics, the rationale for defining genetic parenthood often serves to define the scope of the possible moral implications of genetic parenthood, including parental obligations (Bowman-Smart 2021).

A limitation of this scoping review is that the review only included works that were published in English and Dutch because of the reviewer’s (L.v.R.) language proficiency in these two languages. A further study with a focus on other languages is therefore suggested. Furthermore, diverse perspectives across countries, cultures, and religions were not considered when examining the definitions. The concept of biological parenthood is not only descriptive, as it is shaped by our common experiences of parenthood, societal prejudices, and biases that we inherit or are exposed to (Ettinger 2012). Additionally, intuitions, intentions, and judgments can significantly influence how we perceive this concept (Mertes 2014). Therefore, these perspectives could lead to parental stereotypes which might influence the definitions (Johnson 1999). Moreover, even the most meticulous definitions of biological parenthood may struggle to capture the full picture, as people’s feelings and experiences of biological parenthood may be just as important. Some people may have a deep sense of biological parenthood even if they do not strictly conform to established definitions (Mertes 2014).

## Conclusions

The objective of this scoping review was to map the literature on the definitions of biological parenthood in relation to future ARTs, specifically MRT, IVG, and ectogenesis. The review included 131 eligible studies and showed a lack of consensus on the meaning and definition of biological parenthood. The concept of biological parenthood appears to be a fragmented concept that encompasses genetic, gestational, and postnatal ties. Furthermore, the concept of biological parenthood can be interpreted through different conceptual methods. Although genetic and gestational parenthood can be subsumed under the concept of biological parenthood, these concepts can also be interpreted separately. This variety of interpretations leads to inconsistent determinations of biological parenthood in relation to future ARTs. As a result, biological parenthood is expected to lose its fundamental role in ethical and legal ascriptions of parenthood.

## Supplementary Information

Below is the link to the electronic supplementary material.

Supplementary file1 (XLSX 69 KB)

## Data Availability

Not applicable for this research.

## Footnotes

1 This is indicated by a preliminary search of PROSPERO, PubMed, Web of Science, PsycINFO, HeinOnline (Law Journal Library), Westlaw International, Philosopher’s Index, the Cochrane Database of Systematic Reviews, the Open Science Framework, and *JBI Evidence Synthesis* between July 4**,** 2023 and September 5, 2023.

2 For example, this is the case in the Netherlands (Article 1:198 Dutch Civil Code) and the United Kingdom (Human Fertilisation and Embryology Act 2008s 33 (1)).

3 This literature is drawn from the following of databases: PubMed, PsycINFO, Web of Science, HeinOnline, Westlaw International and Philosopher’s Index. These databases were considered relevant on behalf of the review questions.

4 This is the case, for example, in the Netherlands and the United Kingdom.

5 This is reflected in the types of journals in which the results appear. Of the 131 results, twenty appeared in *Bioethics* and sixteen appeared in the *Journal of Medical Ethics*.
